# Characterization and Strain Improvement of a Hypercellulytic Variant, *Trichoderma reesei* SN1, by Genetic Engineering for Optimized Cellulase Production in Biomass Conversion Improvement

**DOI:** 10.3389/fmicb.2016.01349

**Published:** 2016-08-29

**Authors:** Yuanchao Qian, Lixia Zhong, Yunhua Hou, Yinbo Qu, Yaohua Zhong

**Affiliations:** ^1^State Key Laboratory of Microbial Technology, School of Life Sciences, Shandong UniversityJinan, China; ^2^Shandong Institute for Food and Drug ControlJinan, China; ^3^Bioengineering Institute, Qilu University of TechnologyJinan, China

**Keywords:** *Trichoderma reesei*, uracil auxotrophy, β-glucosidase, biomass conversion, pretreated corncob residues

## Abstract

The filamentous fungus *Trichoderma reesei* is a widely used strain for cellulolytic enzyme production. A hypercellulolytic *T. reesei* variant SN1 was identified in this study and found to be different from the well-known cellulase producers QM9414 and RUT-C30. The cellulose-degrading enzymes of *T. reesei* SN1 show higher endoglucanase (EG) activity but lower β-glucosidase (BGL) activity than those of the others. A uracil auxotroph strain, SP4, was constructed by *pyr4* deletion in SN1 to improve transformation efficiency. The BGL1-encoding gene *bgl1* under the control of a modified *cbh1* promoter was overexpressed in SP4. A transformant, SPB2, with four additional copies of *bgl1* exhibited a 17.1-fold increase in BGL activity and a 30.0% increase in filter paper activity. Saccharification of corncob residues with crude enzyme showed that the glucose yield of SPB2 is 65.0% higher than that of SP4. These results reveal the feasibility of strain improvement through the development of an efficient genetic transformation platform to construct a balanced cellulase system for biomass conversion.

## Introduction

Lignocellulosic biomass, which includes agricultural crop and forest residues, is the most abundant renewable resource on earth (Singhania et al., 2013). It is considered a promising alternative to fossil fuel because it can be converted to second-generation biofuels, such as bioethanol (Gusakov, 2013). However, the relatively high cost of cellulase preparation for cellulose deconstruction is a major obstacle that limits the large-scale commercial production of bioethanol with lignocellulosic biomass as the substrate (Wu et al., 2011). Most of the current cellulase preparations involve the use of filamentous fungi, such as *Trichoderma reesei*, but the ability to produce cellulase through the use of a wild strain is not always fit for industrial requirements (Weiss et al., 2013). Therefore, different well-known hypercellulolytic mutants, such as *T. reesei* QM9414 and RUT-C30, have been developed through classical mutation (Montenecourt and Eveleigh, 1977, 1979). Compared with random mutation, genetic modification provides a rational strategy for strain improvement. However, the main limitation of this strategy is the selection of a proper marker for genetic transformation. Diverse selection mechanisms, including auxotroph complementation (Penttilä et al., 1987), antibiotic resistance (Bailey et al., 2002), and nitrogen utilization (Te’o et al., 2002), have been applied to filamentous fungi. Auxotrophic markers, especially the mutation of the orotidine 5′-monophosphate decarboxylase gene (*pyr4/G* or *ura3*) that leads to uracil auxotrophy, are widely applied in fungal transformation because of their high efficiency and ease in obtaining transformants (Smith et al., 1991). Moreover, a *pyr4* blaster cassette containing a direct repeat sequence can be recycled by screening with 5′-FOA as the counter selection for multiple gene operations (Hartl and Seiboth, 2005). Evidently, construction of a uracil auxotroph strain derived from a cellulase hyperproducing mutant could facilitate genetic modification and strain improvement.

*Trichoderma reesei* is one of the most popular industrial filamentous fungi utilized for cellulase production. Its cellulase system cannot be present in optimal ratios for the deconstruction of lignocellulosic substrates, because it was originally isolated from decayed canvas (Mandels and Reese, 1957; Gomes et al., 1992). The well-known *T. reesei* mutants, including QM9414 and RUT-C30, were generated through random mutation with only cellulose as the screening substrate. Hence, their cellulase preparations are unfeasible for large-scale production and conversion of lignocellulosic materials into bioethanol. Given their capacity to secrete high-level cellulases, *T. reesei* strains still occupy the dominant position as a suitable starting point for the construction of engineered strains used to produce highly active and economically feasible cellulases for the bioethanol industry (Rosgaard et al., 2006). The *T. reesei* cellulase system is composed of endoglucanase (EG) and cellobiohydrolase (CBH), which jointly hydrolyze cellulose to produce cellooligosaccharide and cellobiose (Teugjas and Väljamäe, 2013). These products are further degraded into glucose by β-glucosidase (BGL) to complete the rate-limiting step of cellulose hydrolysis (Sørensen et al., 2013). However, the low amount of secreted BGL (i.e., BGL1) usually leads to incomplete cellobiose hydrolysis, which in turn inhibits the product of the *T. reesei* enzyme system (Rosgaard et al., 2006). Hence, various strategies, such as exogenous addition of BGLs (Duenas et al., 1995), co-cultivation of high-BGL activity strains with *T. reesei* (Gutierrez-Correa et al., 1999), and genetic transformation of *T. reesei* with homologous and heterologous *bgl* genes, have been proposed to supplement BGL activity (Zhang et al., 2010; Ma et al., 2011; Treebupachatsakul et al., 2015).

In our previous work, a hypercellulolytic variant SN1 was isolated on Carboxy Methyl Cellulose (CMC) agar plates and also used for cellulase production. Here, the strain SN1 was identified as *T. reesei* and characterized with high endoglucanase activity. For strain improvement, a uracil auxotroph strain was constructed by deleting the *pyr4* gene via homologous recombination in SN1. The β-glucosidase gene was further overexpressed by using the strong engineered *cbh1* promoter to optimize the cellulase system for the saccharification of different pretreated corncob residues.

## Materials and Methods

### Strains, Plasmids, and Culture Conditions

*Trichoderma reesei* QM9414 (ATCC 26921), which is a general cellulase producer, and RUT-C30 (ATCC 56765), which is another cellulase high-producing strain that is less sensitive to glucose repression, were utilized as the control strains for the comparison of cellulase production. The strain SN1 is a hypercellulolytic variant isolated from our laboratory (Song et al., 2016) and used as the initial host for strain improvement. *Escherichia coli* DH5a (TransGen, Beijing, China) was used for vector construction and propagation. The pMD18-T cloning vector (Takara, Otsu, Japan) was purchased for TA cloning. The pTHB vector was used for *bgl1* overexpression; this vector contained a *T. reesei bgl1* expression cassette under the control of a modified four-copy *cbh1* promoter, as described by Zhang et al. (2010). The plasmid pAB4-1 contained the *Aspergillus niger pyrG* gene as a selection marker to encode orotidine-5′-phosphate decarboxylase (Hartl and Seiboth, 2005). All strains were grown and maintained on a potato dextrose agar plate (PDA) containing 20.0 g/L D-glucose and 20.0 g/L agar for 5–7 days at 30°C. The conidia were harvested, and 10^6^ conidia were inoculated in a 500 mL flask containing 150 mL minimal medium (MM; Penttilä et al., 1987). MM with 300 μg/mL hygromycin B and 1.5 mg/mL 5′-FOA (Sigma, USA) was applied as the selective medium to screen the uracil auxotroph transformants (Singh et al., 2015). An esculin plate containing 3 g/L of esculin, 10 g/L of sodium carboxymethy cellulose (CMC–Na), 0.5 g/L of ferric citrate, and 20 g/L agar was utilized to screen the strains showing β-glucosidase (BGL) activity. A CMC plate containing 10 g/L of CMC–Na, 1 g of yeast extract, and 20 g/L agar was utilized to screen the strains showing cellulase (EG) activity.

### Morphology and Molecular Identification

The fungal strains were grown on PDA plates or solid MM with 2% glucose, 2% glycerol, or 2% lactose as the carbon sources. Photographs of the colonies were obtained with a SONY DSC-HX400 camera for morphology comparison. Microscopic images were captured with a Nikon Eclipse80i light microscope (Nikon, Japan). The fungal genomic DNA was isolated according to the method of Penttilä et al. (1987). For phylogenetic identification of the variant strain SN1, 18S and internally transcribed spacer (ITS) rDNA gene fragments were amplified through polymerase chain reaction (PCR) with the universal primer pairs 18S-F1/18S-R1 and ITS-F1/ITS-R1 (**Table 1**; White et al., 1990), respectively. The SN1 genomic DNA was utilized as the PCR template. The amplified products were purified, sequenced, and compared with the sequences of fungal rDNA 18S and ITS from the NCBI database through the use of the BLAST algorithm. Multiple sequence alignment was conducted with ClustalX. Maximum composite likelihood method analysis was conducted with the MEGA 5 software.

**Table 1:** Primers used in this study.

Primers	Sequences (5′–3′)	Employment
18S-F1	TTCCAGCTCCAATAGCGTAT	Strain identification
18S-R1	CAGACAAATCACTCCACCAAC	Strain identification
ITS-F1	TCCGTAGGTGAACCTGCGG	Strain identification
ITS-R1	TCCTCCGCTTATTGATATGC	Strain identification
pyr4-UF1	AGTGTTTGATGCTCACGCTC	Mutant construction
pyr4-UR1	GGAGATGTTGCTGAAGTCGA GGCGAGGGAGTTGCTTTA	Mutant construction
pyr4-DF1	ATGAGTCGTTTACCCAGAATAAG AAAGGCATTTAGCAAGA	Mutant construction
pyr4-DR1	TGAACAGTAAGGTGTCAGCA	Mutant construction
pyr4-UF2	TGATGCTCACGCTCGGAT	Mutant construction
pyr4-DR2	TCGTCTCGTTCAGCTCGTAATC	Mutant construction
Ypyr4-UF1	ACACAACCTACTGAGCAGAACC	Mutant construction
Yhph-F2	GTCTGGACCGATGGCTGTG	Mutant construction
Hph-F1	CGACTTCAGCAACATCTCC	Mutant construction
Hph-R1	ATTCTGGGTAAACGACTCAT	Mutant construction
Yhph-F1	GCAAAGTGCCGATAAACA	Mutant construction
Yhph-R1	GCGAAGGAGAATGTGAAG	Mutant construction
pyrG-S	CTTCCTAATACCGCCTAGTCAT	Mutant construction
pyrG-A	AGCCGCTGGTCAATGTTATC	Mutant construction
YpyrG-F1	ATCAACACCATGTCCTCCAA	Mutant construction
YpyrG-R1	ACACGAATCCCATAACGAAG	Mutant construction
Y1	GCCAGGGATGCTTGAGTGTA	Mutant construction
Y2	CCCAGCCACAGGACCAAGTATG	Mutant construction
pyr4-probe-F	GCCATCCTCCTTCTTCCTCT	Probe
pyr4-probe-R	TGATACACACAAGTCTGCCAGAT	Probe
bgl-probe-F	TTGAGCCCAATCAGAAATGCGT	Probe
bgl-probe-R	CCCAGCCACAGGACCAAGTATG	Probe


### Construction of the *pyr4* Deletion Strain

The Δ*pyr4*::*hph* cassette for *pyr4* gene disruption was constructed with the double-joint PCR method (Yu et al., 2004). The cassette carried the *E. coli* hygromycin B phosphotransferase (*hph*) gene as the selection marker between the 5′- and 3′-flanking regions of *pyr4*. Specifically, the *pyr4* 5′- and 3′-flanking sequences were amplified with two primer pairs, pyr4-1500-UF1/pyr4-72-UR1 and pyr4-60-DF1/pyr4-1540-DR1 (**Table 1**), respectively, with the SN1 genomic DNA as the template. The hygromycin B phosphotransferase gene (*hph*) was amplified with hph-F1/hph-R1 primers (**Table 1**). The amplicons were then mixed in 1:3:1 molar ratio of 5′-flanking region:hph:3′-flanking region and jointed together during another round of PCR without primers. The resulting PCR product was used directly as a template for the third round of PCR to construct the *pyr4* disruption cassette with the nest primers pyr4-1400-UF2/pyr4-1488-UR2 (**Table 1**). The *pyr4* disruption cassette was transformed into SN1 protoplasts afterward through the method described by Penttilä et al. (1987). The target transformants were screened on MM containing 300 μg/mL hygromycin B and 1.5 mg/mL 5′-FOA. The purified candidate transformants were identified through PCR and Southern blot with the genomic DNA as the template to verify if the *pyr4* locus was replaced by the deletion cassette.

### Overexpression of the β-Glucosidase *bgl1* Gene in *T. reesei* SP4

The vectors pTHB and pAB4-1 were co-transformed into protoplasts of *T. reesei* SN1 through the above mentioned method. The transformants were directly screened on MM. The candidates were then verified through PCR by using primers Y1 and Y2 (**Table 1**), as described by Zhang et al. (2010). Esculin agar plates were prepared and utilized to confirm the *bgl1* overexpression strain.

### Southern Blot Analysis

The probe of *pyr4* was a fragment amplified through PCR by using primers pyr4-probe-F and pyr4-probe-R to detect the *pyr4* gene. The *Xba*I/*Hind*III-digested genomic DNA was hybridized by the *pyr4* probe. The probe of *bgl1* was a fragment amplified through PCR by using primer bgl1-probe-F/bgl1-probe-R to detect the *bgl1* gene. The *EcoR*I-digested genomic DNA was hybridized by the *bgl1* probe. The probe-hybridized DNA fragment was detected with the DIG High Prime DNA Labeling and Detection Starter Kit I (Roche Diagnostics, Mannheim, Germany).

### Shake Flask Cultivation for Cellulase Production

The seed cultures for cellulase production were prepared in MM after incubation at 200 rpm and 30°C for 36 h in a 300 mL flask. Then 15 mL of the activated cells was transferred to 150 mL of the cellulase production medium (CPM) in a 500 mL flask. The CPM composition was as follows (g/L): 20 Microcrystalline cellulose, 2.5 (NH_4_)_2_SO_4_, 5 KH_2_PO_4_, 0.6 MgSO_4_^.^7H_2_O, 1 CaCl_2_^.^2H_2_O, and 20 corn steep liquor.

### Cellulase Activity Assay and Zymogram Analysis of β-Glucosidase Activity

The filter paper (FP), EG, CBH, and BGL activities of *T. reesei* were measured as described before (Ghose, 1987) by using Whatman No. 1 FP (Whatman, UK), CMC–Na (Sigma, USA), *p*-nitrophenyl-β-D-cellobioside (pNPC; Sigma, USA), and *p*-nitrophenyl-β-D-glucopyranoside (PNPG; Sigma, USA) as the substrates, respectively. One unit of enzyme activity was defined as the amount of enzymes releasing 1 mole of reducing sugar (or *p*-nitrophenol in the CBH and BGL assays) per minute under the assay conditions. Renaturing SDS–PAGE electrophoresis was performed in 12% polyacrylamide separating gel containing 0.1% SDS with 0.3% CMC–Na as the substrate; this procedure was performed in a Mini-PROTEAN tetra electrophoresis cell (Bio-Rad Laboratories, Milan, Italy).

### Saccharification of the Pretreated Corncob Residues

Acid-pretreated and delignined corncob residues were used as substrates in the saccharification process; their composition has been described by Zhao et al. (2008) (Supplementary Table S1). The cellulase crude complexes for the saccharification of the pretreated corncob residues were placed in 100 mL flasks containing 5% (w/v) substrate. The pH value and temperature were adjusted to 4.8 (with 50 mM citric acid buffer) and 50°C, respectively. Enzyme loading was adjusted to the same FP activity (10 FPU/g substrates). Glucose production was detected with an SBA-40C biological sensor analyzer (BISAS, Shandong, China) after incubation for 24 or 48 h. Cellulose conversion was calculated as follows:

$$Cellulose\,\;conversion=\frac{Glu\,\cos e\,\;yields\left(mg\right)}{Substrate\,\;weight(mg)\times }\times 0.9\times 100\%\;Cellulose\,\;content\,\;\left(\%\right)$$

## Results

### Molecular and Morphological Identification of Cellulolytic Strain SN1

The cellulase production variant SN1 was identified with partial 18S rDNA and ITS sequences to characterize its phylogenetic relationships. Both the 18S rDNA gene and ITS sequences of SN1 showed 100% identity to those of *T. reesei* (**Supplementary Figure S1**). Hence, the strain SN1 was defined as a variant of *T. reesei*.

**FIGURE 1:**
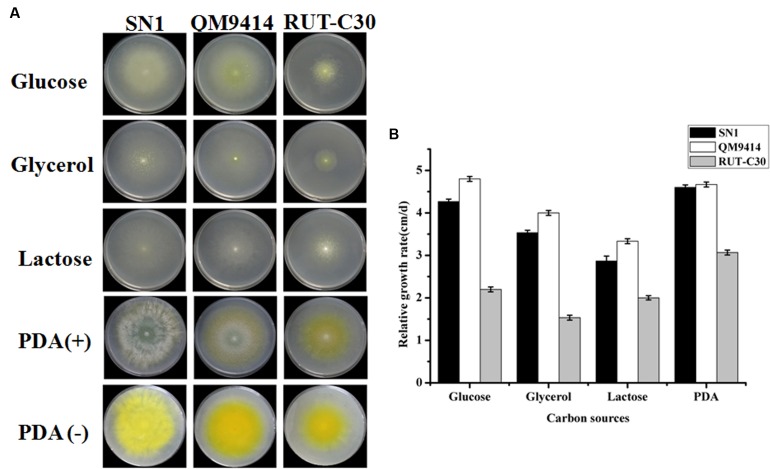
**Growth of *Trichoderma reesei* SN1, QM9414 and RUT-C30 on different carbon sources.** **(A)** Strains were grown on plates containing minimal medium supplemented with 2% glucose, 2% glycerol, 2% lactose, or PDA at 30°C for 3 days. **(B)** The growth was represented by the relative growth rate which was measured as the colony diameter increase per centimeter per day.

*Trichoderma reesei* is a cellulolytic filamentous fungus originally isolated in the Solomon Islands during World War II. *T. reesei* QM9414 and RUT-C30 are among the most well-known high cellulase-producing mutants after 30 years of strain improvements (Peterson and Nevalainen, 2012). They are utilized as control strains in the current study for comparison with SN1 to analyze growth in different carbon sources. All strains were cultured on plates containing 2% glucose, 2% glycerol, and 2% lactose and on PDA plates for 4 days. As shown in **Figure 1A**, SN1 exhibited a colony morphology similar to that of QM9414 and RUT-C30 on glucose, glycerol, and lactose but showed increased radial growth and reduced pigment production on the PDA plates. The growth rate of SN1was comparable to that of QM9414 but higher than that of RUT-C30 on all the growth media tested in this study (**Figure 1B**). Microscopic observations revealed that SN1 had a spore morphology similar to that of the others but possessed a less-branched mycelium (**Supplementary Figure S2**). In summary, the strain SN1 is a novel *T. reesei* variant with high EG activity.

### Cellulase Production and Its Cellulolytic Potential in Biomass Saccharification by *T. reesei* SN1

**FIGURE 2:**
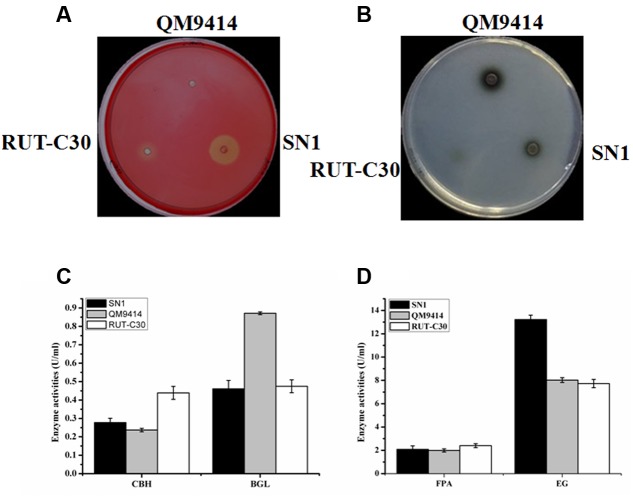
**A comparison of cellulase production in *T. reesei* SN1, QM9414 and RUT-C30.** **(A)** The endoglucanase production of *T. reesei* SN1, QM9414 and RUT-C30 on the condition of 1% CMC-Na with 0.5% triton at 30°C for 2 days. **(B)** Detection of β-glucosidase activity on the CMC-esculin plate in *T. reesei* SN1, QM9414 and RUT-C30 at 30°C for 24 h. **(C)** The FPA and EG activities of the *T. reesei* SN1, QM9414 and RUT-C30, were measured after being induced in cellulase fermentation medium for 5 days. **(D)** The CBH and BGL activities of the *T. reesei* SN1, QM9414 and RUT-C30, were measured as the above mentioned. All results are represented as the mean of three independent experiments; error bars express the standard deviation.

**FIGURE 3:**
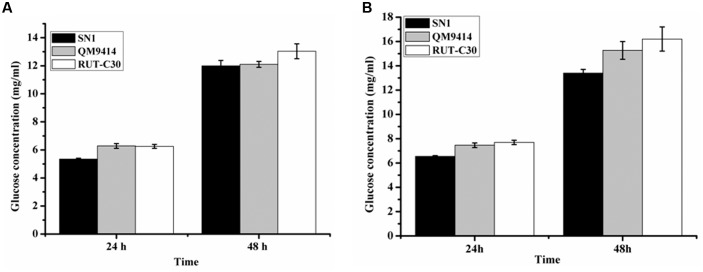
**Saccharification of different pretreated concob residues by *T. reesei* SN1, QM9414 and RUT-C30.** **(A)** Saccharification of acid pretreated corncob residues with equal FPA activity. **(B)** Saccharification of delignined corncob residues. Data are represented as the mean of three independent experiments; error bars express the standard deviations.

To investigate the capacity to secrete cellulase, the fungal strains were cultured on media containing CMC as the substrate, which is generally used to detect EG activity. The media plate was dyed with 1% congo red and decolored with 1 M NaCl after 3 days of cultivation. The cellulolytic halo around the SN1 colony was much larger than those of RUT-C30 and QM9414 (**Figure 2A**). This result indicates that *T. reesei* SN1 probably secreted high-level EG. Another medium containing CMC and esculin as the substrate was applied to test the ability to secrete β-glucosidase (**Figure 2B**). The size of the black halo around the SN1 colony was smaller than that of QM9414, suggesting that *T. reesei* SN1 secreted a relatively low level of BGL. *T. reesei* SN1, QM9414 and RUT-C30 were fermented in CPM to further verify cellulase production. The culture supernatants were also used for the cellulase activity assay. As shown in **Figure 2C**, *T. reesei* SN1 produced a total cellulase activity (FPA) comparable to that of QM9414 but exhibited a notably higher (nearly two times) EG activity than QM9414 and RUT-C30. Comparison of CBH and BGL activities showed that SN1 also produced a CBH activity similar to that of QM9414 but had a much reduced (almost half) BGL activity (**Figure 2D**). These results confirm that *T. reesei* SN1 is a novel hypercellulytic strain that produces prominent EG activity but minimal BGL activity. These results prompted us to examine the saccharification potential of the SN1 enzyme to convert cellulosic materials. The experimental design involved the enzymatic hydrolysis of two types of pretreated corncob residues. This hydrolysis was performed at 50°C and pH of 4.8 for 48 h. In the saccharification of the acid-pretreated corncob residues, the amount of glucose released using the SN1enzyme at 24 h was only 5.3 mg/mL (i.e., 15.2% cellulose conversion), which was lower than the values for QM9414 (6.3 mg/mL corresponding to 17.9% cellulose conversion) and RUT-C30 (6.4 mg/mL corresponding to 17.9% cellulose conversion). The glucose releases in SN1 (12.0 mg/mL corresponding to 34.4% cellulose conversion) and QM9414 (12.1 mg/mL corresponding to 34.8% cellulose conversion) were similar after an additional 24 h of enzymatic hydrolysis (i.e., after a total enzymatic reaction of 48 h). However, they were still lower than that in RUT-C30 (13.3 mg/mL corresponding to 38.4% cellulose conversion; **Figure 3A**). When the delignined corncob residues were used as the substrate, the final glucose release in SN1 (13.4 mg/mL corresponding to 36.7% cellulose conversion) after a 48 h reaction was still lower than those in QM9414 (15.3 mg/mL corresponding to 42.0% cellulose conversion) and RUT-C30 (16.2 mg/mL corresponding to 44.4% cellulose conversion; **Figure 3B**). Therefore, *T. reesei* SN1 exhibited outstanding EG activity. However, its saccharification efficiency for cellulosic materials was obviously lower than those of QM9414 and RUT-C30 probably because of the insufficiency of β-glucosidase in the SN1enzyme system.

### Generation of a *pyr4* Disruption Strain from *T. reesei* SN1

**FIGURE 4:**
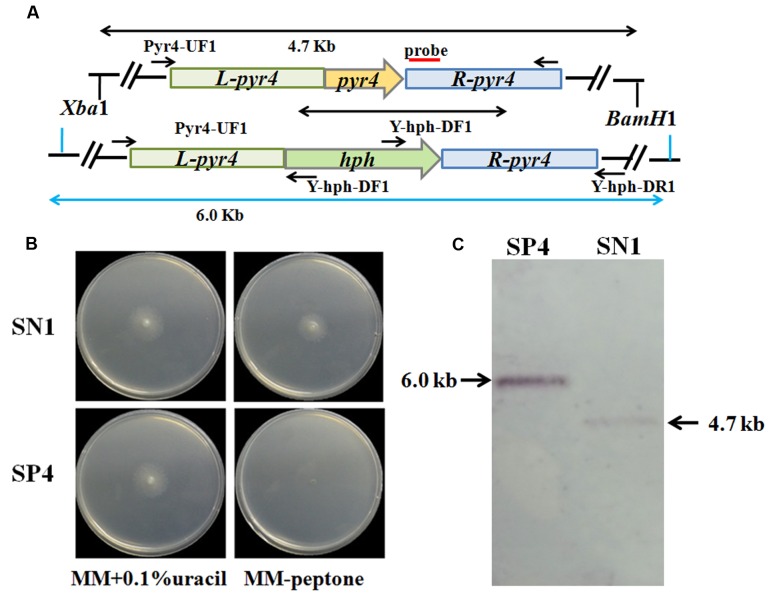
**Deletion of *pyr4* gene in *T. reesei* SN1.** **(A)** Schematic representation of the pyr4 locus in the Δpyr4 and parent strains. Relative positions of the *Xba*I/*Hind*III enzyme restriction sites are noted. Probe used for Southern analysis is shown as red box. **(B)** Phenotypic assay on MM supplemented with uracil (left) and MM without uracil (right) at 30°C for 2 days. **(C)** Southern blot of the genome digested with *Xba*I*/Hind*III. A fragment of 4.7 kb is present in the parent strain, the 6.0 kb band is shown in Δ*pyr4* strains.

The uracil auxotroph strain maximizes high-efficiency genetic transformation and can be used for multiple gene manipulations depending on the *pyr4* marker gene that can be bidirectionally selected (Seidl and Seiboth, 2010). Thus, disruption of the *pyr4* gene in *T. reesei* SN1 to construct the uracil auxotroph strain will contribute to strain improvement through genetic modification. In this study, a Δ*pyr4*::*hph* cassette was constructed and utilized to disrupt the *pyr4* gene in *T. reesei* SN1 (**Figure 4A**). The generated uracil auxotroph strain SP4 was confirmed through plate screening. As shown in **Figure 4B**, SP4 could normally grow on MM containing 0.1% uracil but could not grow on MM without uracil. The *Xba*I/*Hind*III-digested genomic DNA was hybridized with the *pyr4* probe for further Southern blot assay. It yielded a 6.0 kb fragment for the strain SP4 and a 4.7 kb fragment for the parent strain SN1 (**Figure 4C**). These results suggest that the *pyr4* gene was successfully knocked out in SP4. Furthermore, comparison of the cellulase production of SP4 and SN1 showed that the cellulase activities of the SP4 crude enzyme preparations were almost similar to those of SN1 (data not shown).

### Overexpression of *bgl1* under the Control of the Artificial Four-Copy *cbh1* Promoter in *T. reesei* SP4

**FIGURE 5:**
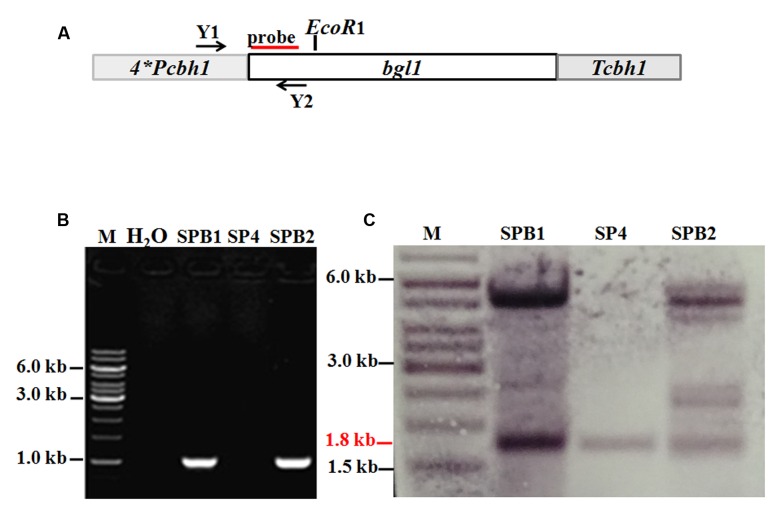
**Overexpression of *bgl1* gene in the Δ*pyr4* mutant.** **(A)** An expression cassette containing the *bgl1* gene is controlled by the *T. reesei* modified *cbh1* promoter. Relative positions of the *EcoR*I enzyme restriction sites are noted. Probe used for Southern analysis is shown as red box. **(B)** PCR assay of the BGL overexpression transformants. Both the transformants SPB1 and SPB2 show a 1.0 kb DNA fragments panning using the primers Y1 and Y2, M: DNA marker. **(C)** Southern blot of the genome digested with *EcoR*1. The 1.8 kb (red line) wild-type band is present in all the strains. The additional bands indicate the *bgl1-*overexpression cassette was successfully integrated into the chromosome.

**FIGURE 6:**
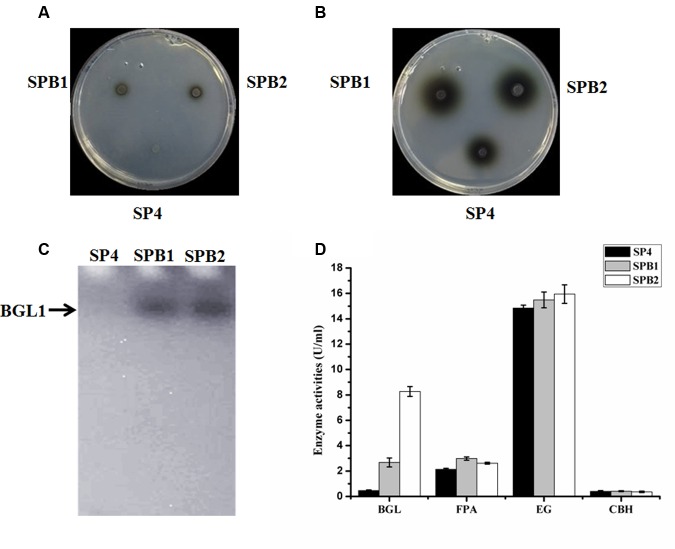
**Phenotypic and cellulase production assay of *bgl1* overexpression transformants.** **(A)** The black halo was observed on the transformants SPB1 and SPB2 at the 12 h. **(B)** The black halos were observed on the transformants SPB1 and SPB2 at the 24 h. **(C)** Detection of β-glucosidase activity from the culture-free supernatant of *T. reesei* SN1, SPB1and SPB2 performed on 12% SDS–PAGE containing 0.5% esculin as substrate. **(D)** BGL, FPA, EG, CBH activities were measured after being induced in cellulase fermentation medium for 5 days. Data are represented as the mean of three independent experiments; error bars express the standard deviations.

To overcome the lack of BGL in the SP4 enzyme system, the pTHB plasmid containing the *bgl1* gene under the control of a strong modified *cbh1* promoter, as described by Zhang et al. (2010) (**Figure 5A**), was co-transformed with the *A. niger pyrG-*containing plasmid pAB4-1 into *T. reesei* SP4. The 126 positive transformants were all further screened on esculin plates to test the BGL secretion (data not shown). This esculin method has been widely used to screen strains showing BGL activity (Lee et al., 2014). Two of the transformants, namely, SPB1 and SPB2, showed much larger black zones around the colony than the parental strain SP4 (**Figures 6A,B**). They were then selected and verified through PCR amplification of the *bgl1* gene using the primers Y1 and Y2 (**Figure 5B**). For further analysis by Southern blot, the *EcoR*I*-*digested genomic DNA was hybridized by the *bgl1* probe and yielded a 1.8 kb fragment for the parent strain SP4 (**Figure 5C**). An additional 5.0 kb band was observed for SPB1, and four additional bands (2.3, 4.5, 5.0, and 5.5 kb) were found for SPB2. These results suggest that one and four *bgl1* copies were successfully integrated into the genomes of SPB1 and SPB2, respectively.

### Characterization of Cellulase Production by the *bgl1-*Overexpression Strains

The *bgl1*-overexpression strains were cultivated in a CPM for 5 days. The culture supernatants were collected for the cellulase activity assay. First, renaturing SDS-PAGE analysis on 12% polyacrylamide gel containing 0.3% esculin as a substrate was applied to detect the BGL secreted by the fungal strains. **Figure 6C** shows a clear band in the secreted proteins of transformant SPB1 or SPB2 but not in SP4. This result indicates that the two *bgl1-*overexpression strains are very likely to secrete a much higher level of BGL than the parental strain. In accordance with this result, the BGL activities of SPB1 and SPB2 measured with pNPG as a substrate were 4.7 and 17.1 times higher than that of SP4, respectively (**Figure 6D**). FP activity (FPA) is generally utilized to evaluate the total cellulase activities. The FPA of SPB1 and SPB2 showed 39.7 and 22.6% increase compared with that of the parent strain SP4 (**Figure 6D**). However, the EG and CBH activities of the two transformants were not significantly changed, besides a relatively lower CBH activity (10.0% decrease) in SPB2 (**Figure 6D**). Interestingly, the highest β-glucosidase activity (8.3 U/mL) was found in SPB2; the value was 3.2-fold higher than that of SPB2 (2.6 U/mL). However, the FPA in SPB2 (2.6 U/mL) was lower than that in SPB1 (3.0 U/mL). This difference in FPA in the two transformants may have been caused by the negative effect of the additional four copies of *cbh1* promoters in the SPB2. It was reported that multiple copies of promoters might result in the titration effect of transcriptional regulators for cellulase gene expression (Rahman et al., 2009; Zhang et al., 2010).

### Saccharification of the Corncob Substrates by Cellulase Preparations from *T. reesei* SPB1 and SPB2

**FIGURE 7:**
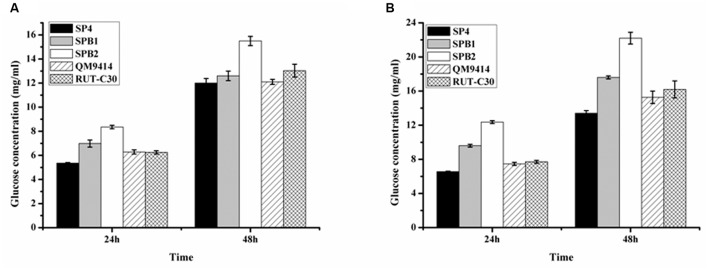
**Saccharification of different pretreated concob residues by the BGL-overexpression strains.** **(A)** Saccharification of acid pretreated corncob residues by *T. reesei* SP4 and the BGL-overexpresion strains SPB1 and SPB2 with equal FPA activity. **(B)** Saccharification of delignined corncob residues *T. reesei* SP4 and the BGL-overexpresion strains SPB1 and SPB2 with equal FPA activity. Data are represented as the mean of three independent experiments; error bars express the standard deviations.

The ability of the cellulase preparations produced by *T. reesei* SPB1 and SPB2 to hydrolyze the pretreated corncob residues was investigated. In the saccharification of the acid-pretreated corncob residues, the glucose release (12.6 mg/mL corresponding to 36.2% cellulose conversion) using the SPB1 enzyme was slightly higher than that for SP4 (12.1 mg/mL corresponding to 34.4% cellulose conversion) but lower than that for RUT-C30 (13.3 mg/mL corresponding to 38.4% cellulose conversion) after a total enzymatic reaction of 48 h (**Figure 7A**). The SPB2 enzyme exhibited the highest glucose release (15.5 mg/mL corresponding to 44.6% cellulose conversion). This result indicates that the high BGL activity in the SPB2 enzyme contributed to the efficient hydrolysis of the acid-pretreated corncob residues. The final glucose yields of SPB1 (17.6 mg/mL corresponding to 48.2% cellulose conversion) and SPB2 (22.2 mg/mL corresponding to 61.0% cellulose conversion) after 48 h reaction were higher than those of SP4 (13.4 mg/mL corresponding to 36.7% cellulose conversion) and RUT-C30 (16.2 mg/mL corresponding to 44.4% cellulose conversion) when the delignined corncob residues were used as a substrate (**Figure 7B**).Therefore, both *bgl1*-overexpression strains (SPB1and SPB2) exhibited better performance than the parental strain SP4 regardless of whether acid-pretreated or delignined corncob residues were used as the substrate. These results suggest that BGL activity has a notable positive correlation with cellulose conversion and indicates that β-glucosidase plays a key role in the enzymatic hydrolysis of corncob residues.

## Discussion

The application of cellulolytic microorganisms to cellulosic biomass bioconversion is considered a potential sustainable approach to develop novel bioproducts, such as biofuels (Premalatha et al., 2015). Diverse cellulolytic microorganisms, including fungi and bacteria, have been isolated from various environments (Jyotsna et al., 2010; Raddadi et al., 2013; Dantur et al., 2015; Premalatha et al., 2015). However, the ability to produce cellulose-degrading enzymes by using wild strains is not always fit for industrial requirements (Weiss et al., 2013). Hence, much effort has been exerted to mutate or engineer potential strains for application in the cellulase industry. In this study, a hypercellulolytic fungus, SN1, was identified as a *T. reesei* variant according to morphological characteristics and ribosomal DNA sequences.

*Trichoderma reesei* was initially isolated from decayed canvas during World War II and is currently one of the most well-known industrial fungi used for cellulase production (Seidl et al., 2008). Several mutants with high cellulase activity, such as NG-14, QM9414, and RUT-C30, have been obtained after more than half a century of strain improvement. Among these mutants, QM9414 and RUT-C30 are the most widely used in cellulase production and as model strains for basic research on cellulase expression (Stricker et al., 2006; Gyalai-Korpos et al., 2011). Therefore, the SN1 strain was compared with these two mutants for hyphal growth and cellulase production in this study. The growth rate of SN1 was comparable to that of QM9414 but was faster than that of RUT-C30. SN1 produced remarkably reduced pigment and sporulated earlier (**Figure 1**). RUT-C30 can produce approximately 1.4 times the total cellulase activity of QM9414 but only 50% of the β-glucosidase activity of QM9414 (Peterson and Nevalainen, 2012). In this study, SN1 showed a total cellulase activity comparable to that of QM9414 and a notably higher (nearly two times) EG activity than both QM9414 and RUT-C30 (**Figure 2**). Considering that our lab studied *T. reesei* for years, and this strain is quite different from QM9414 (carbon catabolite-repressed, containing the full-length *cre1* gene) and RUT-C30 (carbon catabolite-derepressed, containing the truncated *cre1* gene) whether on morphological phenotype or cellulase production, so we further tested whether the SN1 strain was carbon catabolite-derepressed and found that it was carbon catabolite-repressed and contained a complete carbon catabolite repressor gene *cre1* (data not shown). Therefore, we speculated that SN1 was a novel *T. reesei* variant, which was most probably spontaneously mutated from QM9414 due to the cellulose substrates used as the continued screening pressure. Many studies have shown that EGs can be widely used in industrial activities, such as pulp beatability (Kamaya, 1996), deinking (Gübitz et al., 1998), and bio-polishing (Li et al., 2011). The high-level endoglucanase production showed that SN1 is a potential strain for industrial cellulase production.

However, despite the feature of SN1 cellulases, the respective activities in the cellulase products appeared to be present in suboptimal ratios for lignocellulose degradation. Hence, improvement of the cellulolytic capacity of *T. reesei* SN1 is required. This enhancement is often accomplished through genetic engineering. Auxotrophy selection markers, such as uracil auxotrophy, have been proven highly efficient for *T. reesei* genetic transformation (Hartl and Seiboth, 2005; Steiger, 2013). *T. reesei* TU6 is an auxotrophic strain widely used for its high transformation efficiency (Gruber et al., 1990). However, TU6 was obtained through random γ-radiation mutagenesis from QM9414, resulting in slow growth, decreased conidiation, and reduced cellulase production. Therefore, a new uracil auxotrophic strain, SP4, was constructed in this study through targeted gene deletion from a hypercellulolytic strain (SN1). No discernible difference in growth, conidiation, or cellular morphology was observed between SP4 and its parental strain SN1 (data not shown). Furthermore, SP4 produced a high cellulase level and could be genetically transformed with high efficiency because of the uracil auxotroph. Therefore, *T. reesei* SP4 provides a platform strain suitable for genetic manipulation to improve cellulolytic efficiency in biomass conversion.

The major challenges in the bioconversion of lignocellulosic biomass include obtaining the optimum amount of BGL to complete the final step of cellulose hydrolysis (Singhania et al., 2013; Pryor and Nahar, 2015). Low β-glucosidase activity results in incomplete cellobiose hydrolysis, which further leads to product inhibition in the cellulolytic enzymes. Construction of engineering strains with high BGL activity is the preferred strategy to address this problem. Indeed, studies have shown that overexpression of BGL-encoding genes in *T. reesei* could result in improved BGL activity; this overexpression was mostly accomplished through genetic transformation with hygromycin B resistance (Liu et al., 2013). Wang and Xia (2011) constructed a *T. reesei* strain with a BGL activity of 5.3 U/mL by using the heterologous expression of the *A. niger*β-glucosidase (cellobiase) gene. Ma et al. (2011) reported that heterologous expression of the *Penicillium decumbents*β-glucosidase gene via *Agrobacterium*-mediated transformation in *T. reesei* leads to an eightfold increase in BGL activity. In another study, Zhang et al. (2010) utilized a modified *cbh1* promoter to overexpress the native *bgl1* gene in *T. reesei* and obtained a pyrithiamine-resistant strain with 5.7-fold higher BGL activity. In this study, the same *bgl1* expression cassette was transformed into SP4 with uracil auxotrophy as a selection marker. This uracil auxotroph transformation system showed high efficiency. One transformant, SPB2, exhibiting a 17.1-fold higher BGL activity with additional four copies of *bgl1* gene was selected from 126 positive transformants. The ratio of BGL activity to FPA in SPB2 reached 2.8, which was much higher than that in the parental strain (0.2). Chen et al. (2008) reported that cellobiose would be efficiently degraded into glucose if the ratio of BGL activity to FPA is more than 0.3. Thus, the enzyme system produced by the *bgl1-*overexpression strains constructed in this study can be directly applied to the saccharification of lignocellulosic materials without adding extra BGL.

The SPB2 enzyme containing the highest BGL activity displayed excellent performance in the saccharification of both corncob substrates (**Figure 7**). The SPB1 enzyme was more effective than SP4 in the saccharification of the delignined corncob residues, but both had a similar effect on the hydrolysis of the acid-pretreated corncob residues. A possible reason for the difference maybe that a small amount of β-glucosidase in the enzyme complex was adsorbed on to the lignin component in the acid-pretreated corncob residues, which is in accordance with the postulation of Haven and Jørgensen (2013). The enhancement effect of excessive BGL on the saccharification of cellulosic materials (shown by the SPB2 enzyme) has been previously reported (Peterson and Nevalainen, 2012). Such results can be explained by the possibility that a large amount of β-glucosidase would reach the saturated adsorption of lignin and further degrade cellobiose, thus helping alleviate product inhibition and facilitate cellulose conversion.

## Conclusion

A novel hypercellulolytic *T. reesei* variant, SN1, was characterized. A uracil auxotrophic strain, SP4, was constructed through gene deletion to achieve high-efficiency genetic transformation. Further overexpression of the *bgl1* gene in SP4 significantly enhanced the BGL activity in the cellulolytic enzyme complex and was proven to be highly effective in the saccharification of the pretreated corncob residues. These results reveal the feasibility of strain improvement from a mutant industrial strain via ab initio genetic manipulation. This strategy appears to be practicable as an essential step in the construction of genetically engineered strains for the production of commercially viable enzyme preparations for biomass conversion.

## Author Contributions

YuQ, YH, YiQ, and YZ designed and coordinated the study and wrote the manuscript. YuQ and LZ carried out the experiments and analyzed the results. All authors read and approved the final manuscript.

## Conflict of Interest Statement

The authors declare that the research was conducted in the absence of any commercial or financial relationships that could be construed as a potential conflict of interest.

## Supplementary Material

**FIGURE S1 | Phylogenetic tree of strain SN1 based on the 18S rDNA gene sequence and internally transcribed spacer (ITS) region.** **(A)** 18S rDNA sequences. The 18s rDNA gene sequences from *Trichoderma reesei* (XM_006965206.1), *T. sp* (AB923813.1), *T. harzianum* (KP133166.1), *T. viride* (AF218788), *Penicillium oxalicun* (JQ349066.1), *Aspergillus ni*ger (AM270052.1), *Cladonia arbuscula* (KT148631.1). **(B)** ITS region sequences, the ITS region sequences from *T. reesei* (KT336514.1), *T. harzianum* (KR232487.1), *T. viride* (DQ442274.1), *T. saturnisporum* (KF294853.1), *T. asperelloides* (KT426897.1), *T. pseudokoningii* (AF140046), *P. oxalicun* (KT189516.1), *A. niger* (KM613139.1).

**FIGURE S2 | Mycelium morphology of SN1, QM9414, and RUT-C30.** All strains were cultivated 2–5 days on PDA plates (glucose) to analyze the microscopic morphology of mycelium and spore. The images were captured using DIC. Scale bars are listed 20 μm in the figure.
