# Supplementary figures and images for: Characterization and Strain Improvement of a Hypercellulytic Variant, Trichoderma reesei SN1, by Genetic Engineering for Optimized Cellulase Production in Biomass Conversion Improvement
Source: Front Microbiol. 2016 Aug 29;7:1349. doi: 10.3389/fmicb.2016.01349 (PMC5002442; doi:10.3389/fmicb.2016.01349)

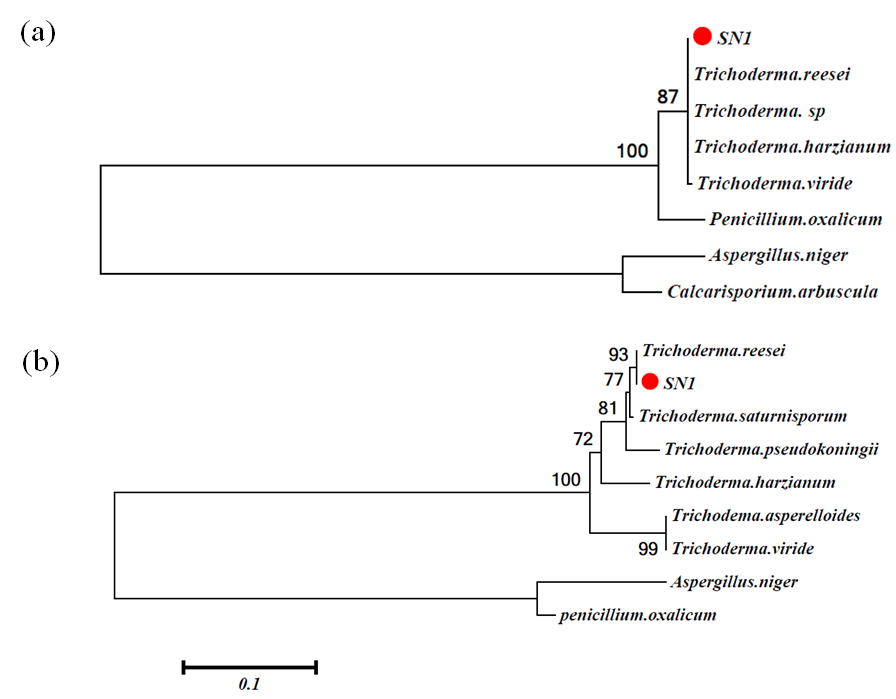

Supplement: Supplementary file 1 [file Image_1.TIF]

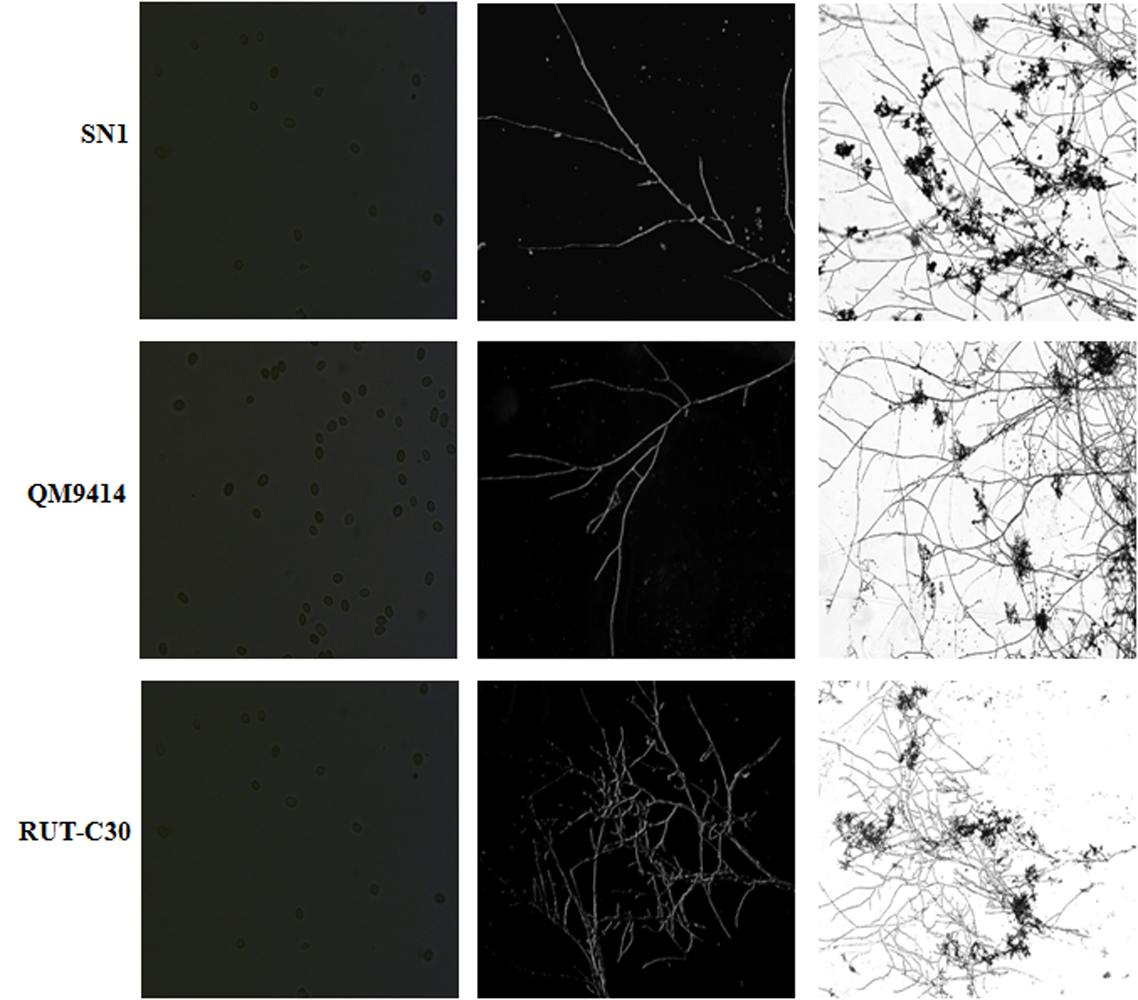

Supplement: Supplementary file 2 [file Image_2.TIF]
