# Supplementary material for: Characterization and Strain Improvement of a Hypercellulytic Variant, Trichoderma reesei SN1, by Genetic Engineering for Optimized Cellulase Production in Biomass Conversion Improvement
Source: Front Microbiol. 2016 Aug 29;7:1349. doi: 10.3389/fmicb.2016.01349 (PMC5002442; doi:10.3389/fmicb.2016.01349)
Supplement: Supplementary file 3 [file Table_1.DOCX]

**Table S1. Composition of cellulose, hemicellulose, lignin and ash in percentage of the pretreated corncob residues**

| Corncob | Cellulose  (%) | Hemicellulose  (%) | Lignin (%) | Ash (%) | Etc.  (%) |
| --- | --- | --- | --- | --- | --- |
| Corncob | 38.50 | 36.40 | 13.1 | 3.20 | 8.50 |
| Acid-pretreatedcorncob | 62.60 | 2.40 | 17.70 | 6.80 | 10.50 |
| Delignined corncob | 65.70 | 1.80 | 3.00 | 5.90 | 23.50 |
